# Patient-specific hemodynamic simulation for left ventricular assist device via non-invasive monitoring based on lumped parameter model and hierarchical neural network

**DOI:** 10.3389/fphys.2026.1738196

**Published:** 2026-04-07

**Authors:** Jiayi Lyu, Xiaoyu Liu, Zhihong Lin, Baohua Ji, Qi Gao, Qiang Shu, Xiangming Fan

**Affiliations:** 1 Transvascular Implantation Devices Research Institute, Hangzhou, China; 2 Institute of Fluid Engineering, School of Aeronautics and Astronautics, Zhejiang University, Hangzhou, Zhejiang, China; 3 State Key Laboratory of Transvascular Implantation Devices, Zhejiang University, Hangzhou, Zhejiang, China; 4 Institute of Biomechanics and Applications, Department of Engineering Mechanics, Zhejiang University, Hangzhou, China; 5 Department of cardiac surgery, Children’s Hospital, Zhejiang University School of Medicine, National Clinical Research Center for Child Health, Hangzhou, Zhejiang, China

**Keywords:** deep learning, heart failure, hemodynamics, left ventricular assist device, lumped parameter model

## Abstract

**Introduction:**

Left ventricular assist devices (LVADs) are widely used in advanced heart failure, but require accurate hemodynamic assessment for optimal management. Current invasive methods such as right-heart catheterisation (RHC) are limited in routine use, highlighting the need for non-invasive alternatives.

**Methods:**

A non-invasive framework combining a lumped parameter model (LPM) with a hierarchical neural network (CLPM-Net) was developed to estimate patient-specific hemodynamic parameters from echocardiography and blood pressure. Model identifiability analysis was performed to select key parameters. The model was trained on synthetic data and validated with clinical cases.

**Results:**

The proposed method achieved accurate parameter estimation with errors below 10% (RMSE). Simulated hemodynamic indicators showed strong agreement with ground truth (nMED < 1%). Clinical validation demonstrated close consistency with invasive measurements.

**Discussion:**

This framework enables non-invasive, patient-specific hemodynamic assessment for LVAD management. It shows potential as an alternative to invasive monitoring, though further large-scale clinical validation is required.

## 1 Introduction

For patients with advanced heart failure (HF), therapeutic options are limited to heart transplantation or left ventricular assist device (LVAD) implantation (Miller et al., 2019). Despite recent increases in the number of donor hearts, the demand still vastly exceeds the supply (Miller et al., 2019). As a result, LVAD has emerged as the primary treatment modality for advanced HF. It provides opportunities for improving quality of life and prolonging survival for patients who are unable to undergo heart transplantation.

Despite substantial advances in third-generation LVADs for optimizing hemodynamics, their long-term implantation can still cause adverse hemodynamic disturbances. These disturbances can lead to hemodynamic-related events (HDREs) such as right heart failure (RHF) and aortic insufficiency (Grinstein et al., 2023). Optimizing LVAD speed through ramp testing can mitigate these adverse effects and substantially reduce hospital readmission rates (Imamura et al., 2019). However, the gold standard for evaluating these hemodynamic changes is invasive right-heart catheterisation (RHC), which is time-consuming, costly, and requires hospitalisation. Its routine clinical use is therefore limited (Rosenkranz and Preston, 2015). In contrast, echocardiography offers a noninvasive, convenient, dynamic, and repeatable assessment that does not require hospitalisation. Yet its objectivity and scientific rigour are often insufficient to replace RHC (Keshavarz-Motamed, 2020). Therefore, there is an urgent need for a new noninvasive and objective method for hemodynamic assessment.

The lumped parameter model (LPM) is a widely adopted computational approach in cardiovascular simulations. It offers an effective balance between efficiency and accuracy by representing the circulatory system’s vessel resistance, blood inertia, and vessel compliance through equivalent circuit elements. These LPMs can be broadly classified into non-specific and patient-specific types. Non-specific LPMs are commonly used to simulate surgical procedures (Bonnemain et al., 2013; Ahmad Bakir et al., 2018; Fu et al., 2020; Neidlin et al., 2016), disease mechanisms (Liang et al., 2014; Tang et al., 2020), and LVAD control strategies (Shi and Korakianitis, 2018; Mehmood et al., 2023). Neidlin et al. (2016) evaluated the availability of the subclavian artery as an LVAD outflow graft site. Liang et al. (2014) analysed hemodynamics in the Fontan procedure. And Shi and Korakianitis (2018) employed LPMs to determine the ideal rotational speed for the HeartMate 3, minimising ventricular suction and regurgitation risks. As for the application of patient-specific LPMs, Bahadormanesh et al. (Keshavarz-Motamed, 2020) proposed an ultrasound-based method to evaluate patients’ hemodynamic indicators non-invasively, while simulating the valve dynamics of transcatheter aortic valve replacement (TAVR) (Bahadormanesh et al., 2023a) and Aortic stenosis (AS) (Bahadormanesh et al., 2023b) surgeries. In general, patient-specific LPMs provide enhanced clinical relevance by more accurately replicating individual hemodynamic responses under pathological conditions. The direct comparability with clinical measurements further guarantees their reliability for diagnostic and therapeutic decision-making.

Currently, the process of patient-specific parameters optimization of LPMs follows these steps: First, a generic LPM framework is constructed, typically using parameter values recommended in the literature. Second, invasive hemodynamic indicators are used as targets, and optimization methods such as the Levenberg-Marquardt (LM) algorithm (Zhang et al., 2020; Casas et al., 2017; Tomasi et al., 2020), gradient descent (Laubscher et al., 2023), or neural network (NN) (Naghavi et al., 2024; Grayson Tong et al., 2024) are applied to estimate the LPM parameters that best match these targets. Researchers like Zhang et al. (2020), Casas et al. (2017), and Tomasi et al. (2020) have employed the LM method to estimate LPM parameters for either the full circulatory system or subsystems such as the left heart and systemic circulation. However, this approach is sensitive to initial parameter choices. If the starting values are far from the global optimum, the optimization may become trapped in local minima (Transtrum and Sethna, 2012; Gavin, 2019). Additionally, the LM method and the gradient descent method require repeated LPM simulations during optimization to ensure convergence, thereby increasing computational time. Many of these studies lack model identifiability analysis (MIA) (Zhang et al., 2020; Tomasi et al., 2020). This means different parameter sets could yield identical simulation results, resulting in non-unique solutions. Alternative methods, such as Physics-Informed Neural Networks (PINNs), incorporate the governing equations of LPMs into the NN’s loss function to ensure mathematical and physical consistency (Naghavi et al., 2024). While this guarantees theoretically valid results, the complexity of the equations limits the sophistication of the LPM structure. For example, the LPM developed by Naghavi et al. (2024) includes only five compartments (left atrium, left ventricle, aorta, peripheral arteries, and vena cava), while Grayson Tong et al. (2024) constructed a six-compartment model (left ventricle, right ventricle, systemic arteries, systemic veins, pulmonary arteries, and pulmonary veins). Such simplifications restrict the model’s ability to monitor some critical hemodynamic indicators like pulmonary artery pressure (PAP) and pulmonary capillary wedge pressure (PCWP).

**FIGURE 1:**
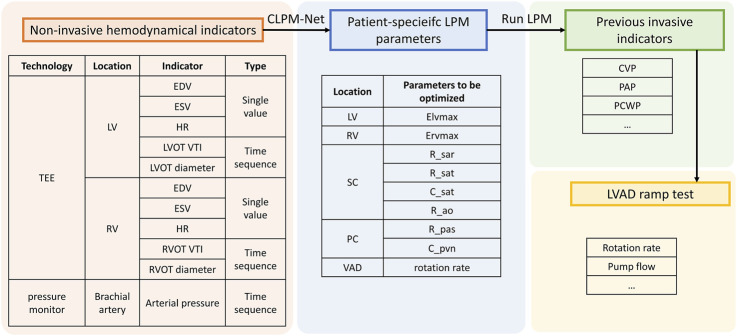
The implementation and application pipeline of this work.

Although Artificial Intelligence (AI)-based approaches such as PINNs still face challenges, they demonstrate high potential for integrating non-invasive measurements with patient-specific LPM. Thus, this work aims to propose a non-invasive data-driven hierarchical neural network for optimizing patient-specific LPM parameters and achieving accurate hemodynamic monitoring in LVAD patients. The novelty of this work includes the following aspects: 1) conducting the first MIA of the LPM for the complete cardiovascular system; 2) proposing a hierarchical NN that combines physics-constrained and data-driven approaches to predict LPM parameters that are relevant to the hemodynamics of LVAD-implanted patients, which is not specific to any particular LVAD model; 3) being the first to apply patient-specific LPM to LVAD-related hemodynamic management, offering a non-invasive, economical, and convenient approach compared to traditional RHC. The procedure is as follows (see Figure 1): obtain cardiac indicators and arterial pressure through non-invasive ultrasound and blood-pressure monitoring; input these values into a well-trained NN (called cardiac-LPM Net, CLPM-Net) to generate patient-specific LPM parameters. Based on this patient-specific LPM, various clinical applications can be conducted, including non-invasive hemodynamic management, LVAD surgical evaluation and simulation of LVAD ramp tests. Therefore, this framework may serve as a potential tool for future clinical application.

## 2 Methods

Existing research has established that data obtained from ultrasound and blood-pressure sensing can be used for continuous calculation of patient-specific cardiovascular parameters (Casas et al., 2017; Bahadormanesh et al., 2023b; Garber et al., 2023; Feng et al., 2024). Building on this, a patient-specific hemodynamic computational framework integrating Transesophageal Echocardiography (TEE) and pressure monitor (as shown in Figure 1) was proposed, aiming to non-invasively obtain LVAD-related hemodynamic indicators that traditionally require invasive techniques. This framework is realised through an LPM of the complete cardiovascular system and a hierarchical NN, CLPM-Net, which integrates both physics-constrained and data-driven approaches. Given the challenges in acquiring clinical data, CLPM-Net was first trained and tested on synthetic data before being validated on three clinical cases.

### 2.1 Basic LPM

**FIGURE 2:**
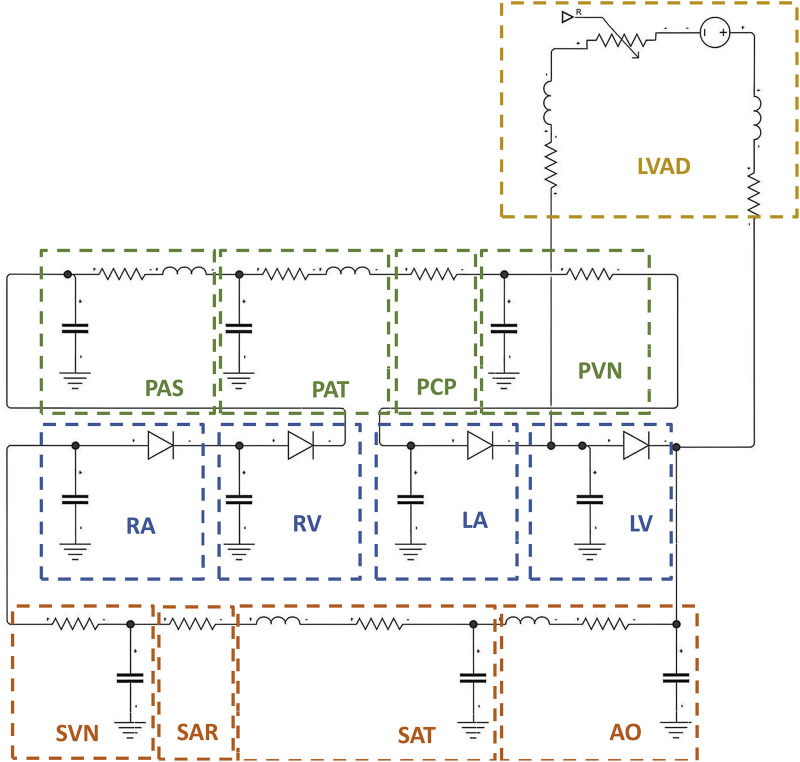
The architecture of the basic LPM of the complete cardiovascular system and LVAD. LVAD: left ventricular assist device, PAS: pulmonary artery sinus, PAT: pulmonary artery, PCP: pulmonary capillary, PVN: pulmonary vein, RA: right atrium, RV: right ventricle, LA: left atrium, LV: left ventricle, AO: aorta, SAT: systemic artery, SAR: systemic arterioles, SVN: systemic vein.

Before patient-specific optimization, a basic LPM is first constructed. Figure 2 illustrates the basic LPM developed in this study, which comprises four components: the heart, systemic circulation, pulmonary circulation, and the LVAD.

#### 2.1.1 Heart

The heart consists of four chambers, each taken as a variable-compliance chamber modelled by a capacitor. Four heart valves (equivalent to four diodes) allow the unidirectional blood flow. During a cardiac cycle, the four chambers alternately contract and relax. The elastance function E(t) is defined as the reciprocal of the capacitance value C(t) (see Equation 2). Throughout the following discussion, E(t) consistently denotes this inverse relationship, represented by a waveform with the cardiac cycle T as its period (Fu et al., 2020). The $E_{\max}$ represents the peak value of E(t) , which occurs at the end-systolic phase and signifies the maximum contractile strength of the heart. An increased $E_{\max}$ reflects enhanced contractility during ejection. By modifying $E_{\max}^{LV}$ and $E_{\max}^{RV}$ , it is possible to model different severity levels of HF. The functions E(t) and $E_{\max}$ are expressed in Equations 1, 2. The total blood volume (TBV) is represented by the sum of the initial charges of the four capacitances.

$$E\left(t\right)=\frac{1}{C\left(t\right)}=\frac{P\left(t\right)}{V\left(t\right)-V_{0}}\;,$$ (1)

$$E_{\max}=\frac{P_{es}}{V_{es}-V_{0}}\;.$$ (2)

Where P(t) is the chamber pressure and $P_{es}$ represents its value at the end of systole. V(t) is the chamber volume, and $V_{es}$ represents its value at the end of systole. $V_{0}$ is the theoretical volume at zero pressure. Basic LPM parameters can be found in Table 1.

**TABLE 1:** Parameter values of basic LPM model. TBV: total blood volume, LVAD: left ventricular assist device.

Location		$E_{\max}$ (mmHg/mL)	$E_{\min}$ (mmHg/mL)	$V_{0}$ (mL)
RA		0.25	0.15	0
RV		1.15	0.07	10
LA		0.25	0.15	0
LV		2.5	0.05	5
TBV (initial charge)			900	
T			0.8	

		R (mmHg ⋅ s/mL)	C (mL/mmHg)	L (mmHg ⋅ ${\text{s}}^{2}$ /mL)
Systemic circulation	Aortic	0.003	0.008	0.0000168
	Arterial	0.05	1	0.0017
	Artery	1.02	–	–
	Vein	0.075	20.5	–
Pulmonary circulation	Aortic	0.002	0.18	0.000052
	Arterial	0.05	3.8	0.0017
	Capillary	0.05	–	–
	Vein	0.006	20.5	–
LVAD	Inlet	0.0677	–	0.0127
	Outlet	0.0677	–	0.0127
	R	−3.5/1	–	–

#### 2.1.2 Systemic and pulmonary circulation

The systemic circulation is divided into four parts (as shown in Figure 2): the aorta, systemic arteries, systemic arterioles, and systemic veins. The pulmonary circulation is also divided into four parts: pulmonary arterial sinus, pulmonary arteries, pulmonary capillaries, and pulmonary veins. Each part is represented by a combination of resistance, capacitance, and inductance based on its respective physiological characteristics.

#### 2.1.3 Left ventricular assist device

In Figure 2, the inlet of the LVAD is connected to the posterior of the mitral valve, while the outlet is connected to the ascending aorta. The LVAD pump is modelled as an ideal voltage source, with the pressure difference H represented by the pump speed w , flow rate Q , and three coefficients $K_{a},K_{b},K_{c}$ (see Equation 3). The w serves as the manipulated variable, while the Q and the pressure head H are the response variables. Different LVADs have distinct $K_{a},K_{b}$ , and $K_{c}$ . This study specifically focuses on equivalent modelling of the CorHeart 6 and HeartMate 3 (as shown in Table 2). The coefficients for the HeartMate 3 are sourced from the literature (Shi and Korakianitis, 2018), while those for the CorHeart 6 are derived from its published pressure-flow curve (Fang et al., 2023). The LVAD pump is connected at both ends by conduits, meaning that the resistance and blood inertia in both conduits cannot be overlooked. These are represented by a pair of resistors and inductors (Chen et al., 2006), respectively. Additionally, a variable resistor element is used to simulate suction conditions (Chen et al., 2006).

$$H=P_{\mathrm{out}}-P_{in}=K_{a}Q^{2}+K_{b}Qw+K_{c}w^{2}.$$ (3)

**TABLE 2:** The parameters $K_{a}$ , $K_{b}$ , and $K_{c}$ of the HeartMate 3 and CorHeart 6.

LVAD	$K_{a}$ (mmHg ⋅ ${\text{s}}^{2}$ /mL^2^)	$K_{b}$ (mmHg ⋅ s/(mL ⋅ rpm))	$K_{c}$ (mmHg/rpm^2^)
HeartMate 3	$-1.8\times 10^{-3}$	$-1.2\times 10^{-5}$	$7.3\times 10^{-6}$
CorHeart 6	$4.82\times 10^{-3}$	$6.66\times 10^{-5}$	$7.2\times 10^{-6}$

#### 2.1.4 Simulation settings

The LPM is numerically solved in MATLAB R2023b Simulink. The simulation employs the variable-step solver ode15s with a relative tolerance of 1e-3. The step size is bounded by a maximum of auto and a minimum of 1e-5. Each run proceeds for 60 s to reach a hemodynamic steady state.

### 2.2 Model identification analysis

The patient-specific optimization of LPM can be classified as a parameter estimation task, which is the inverse process of LPM calculation. It aims at deducing LPM parameters from observed values, which include non-invasive ultrasound metrics and blood pressure. In this study, the LPM parameters fall into four categories, as listed in Table 3.

**TABLE 3:** Categorization of parameters, along with their sources and whether they are patient-specific and predicted by CLPM-Net.

Category	Content	Source	If it is patient-specific (namely, variable)	If it is predicted by CLPM-Net
(1)	Decided by TSF and GSF	System and pulmonary circulation	Yes	Yes
(2)	$E_{lv}^{\max}$ , $E_{rv}^{\max}$	Cardiac	Yes	Yes
(3)	T , TBV	Patient metrics	Yes	No
(4)	Others	Others	No	No

The parameter category (2) contains $E_{LV}^{\max}$ and $E_{RV}^{\max}$ . These two parameters are critical, as they primarily reflect the differences in myocardial contractility between HF patients and healthy individuals. The parameter category (3) encompasses the cardiac cycle T and total blood volume TBV . There is significant inter-individual variability in T , and it plays an essential role in hemodynamic assessment. TBV is also included as a variable due to its substantial individual variability. TBV is derived from body surface area (BSA), which is calculated from height and weight.

The following context introduces the parameter category (1). The complexity of the systemic and pulmonary circulation components presents significant challenges in uniquely determining all parameter values from limited observed values. To address this issue, MIA is conducted. It aims to identify critical parameters that are sensitive to the target scenario, while the remaining parameters are fixed at physiologically reasonable default values. The primary objective of this study is to optimize LPM parameters for LVAD scenarios, rather than pursuing a general optimization of LPM parameters. Consequently, the focus is placed on hemodynamic indices that are directly linked to LVAD assessment: cardiac output (CO), arterial pressure (AP), and PAP (Bouchez et al., 2019; Mehmood et al., 2023). Through MIA, the ability of the estimated parameters to uniquely determine the target indices (CO/AP/PAP) is evaluated. If a single set of target indices corresponds to multiple parameter combinations, it indicates model unidentifiability. The optimization space needs to be refined through sensitivity analysis or parameter dimensionality reduction.

Although the input data for this study includes non-invasive ultrasonography and blood pressure, directly using these raw signals as targets for MIA may result in model parameters that do not align with clinical decision-making priorities. Therefore, CO, AP, and PAP are utilised as target indicators for MIA, ensuring physical consistency between the model output parameters and the LVAD control objectives. Currently, the primary tools employed for MIA include traditional sensitivity analysis and generalized sensitivity analysis (Pant et al., 2014; Thiel et al., 2025).

#### 2.2.1 Traditional sensitivity function

Traditional sensitivity function (TSF) reveals the sensitivity of target indices to changes in estimated parameters. If the target indices are not very sensitive to changes in certain LPM parameters, it is recommended that these parameters be fixed to default values. The definition of TSF is as follows:

$$s_{i,j}\left(t,\theta_{0}\right)=\frac{\partial h_{i}\left(t,\theta_{0}\right)}{\partial \theta_{j}},$$ (4)

where $s_{i,j}\left(t,\theta_{0}\right)$ represents the change in the target indices $h_{i}\left(t,\theta_{0}\right)$ with respect to a small perturbation $\partial \theta_{j}$ of the parameter value. Here, i and j are the indices of the target indices and the estimated parameters, respectively. t indicates that the model is dynamic, and $\theta_{0}$ denotes the baseline value of the estimated parameters, using the non-specific values in Table 1. Due to the different magnitudes of the parameters, a dimensionless treatment of the TSF is typically applied for better comparison, as shown in Equation 5.

$$S_{i,j}\left(t,\theta_{0}\right)=\left(\frac{\theta_{j}^{r}}{h_{i}^{r}}\right)\frac{\partial h_{i}}{\partial \theta_{j}}\left(t,\theta_{0}\right).$$ (5)

Where $\theta_{j}^{r}$ and $h_{i}^{r}$ are the reference values for $\theta_{j}$ and $h_{i}$ . $\theta_{j}^{r}$ is from Table 1 and $h_{i}^{r}$ is computed based on $h\left(t,\theta_{0}\right)$ . A smaller TSF indicates that the target index is less sensitive to the estimated parameter, meaning that the target index contains less information about this parameter. The TSF can be used to reduce the number of estimated parameters: by ranking the sensitivity of all LPM parameters to the three target indices from high to low, the top few LPM parameters can be selected as the estimated parameters, while the others can be fixed.

#### 2.2.2 Generalized sensitivity function

The TSF examines the changes in target indices relative to the estimated parameters. However, the information TSF provided is limited because it overlooks the correlations between the parameters. The generalized sensitivity function (GSF) can serve as a complement to the TSF to address this issue. For inverse problems, the GSF provides (a) the variation over time of the information each parameter contributes to the target indices, (b) the degree of correlation between model parameters, and (c) indications regarding the potential identifiability of the model. The definition of the GSF is as follows:

$$g_{k}\left(t_{n}\right)=\overset{n}{\underset{i=1}{\sum }}\overset{M}{\underset{j=1}{\sum }}\left(\frac{1}{\sigma_{j}^{2}\left(t_{i}\right)}{\left(F^{-1}\times \nabla_{\theta }h_{j}\left(t_{i}\theta_{0}\right)\right)}_{k}•{\left(\nabla_{\theta }h_{j}\left(t_{i}\theta_{0}\right)\right)}_{k}\right),$$ (6)

where $g_{k}$ represents the GSF of the estimated parameter k , $t_{n}$ denotes the observation time (n=1,2,…N) , i is the index for the time points (i=1,…n) , and j is the index for the target indices ( j=1,2,…M , here M=3 ). θ refers to the Gaussian noise. Other symbols are defined similarly to those in Equation 5. $F^{-1}$ represents the Fisher Information Matrix (FIM). The FIM is a value that does not change over time and is defined in Equation 7 as follows:

$$F=\overset{N}{\underset{i=1}{\sum }}\overset{M}{\underset{j=1}{\sum }}\frac{1}{\sigma_{j}^{2}\left(t_{i}\right)}\left(\nabla_{\theta }h_{j}\left(t_{i},\theta_{0}\right)\right){\left(\nabla_{\theta }h_{j}\left(t_{i},\theta_{0}\right)\right)}^{T}.$$ (7)

In the Equation 7, all symbols are defined similarly to those in Equation 6. From Equation 6, it can be seen that the GSF is a cumulative function that includes the contributions accumulated from the initial time to the time $t_{n}$ . The change in the GSF, represented by $\Delta g\left(t_{n}\right)=g\left(t_{n}\right)-g\left(t_{n-1}\right)$ , quantitatively indicates the amount of information the target indices provide about the estimated parameters. A sharp increase in the GSF signifies that the information regarding the estimated parameters is highly concentrated during the corresponding period. When the GSF approaches monotonicity, it can be inferred that the parameter is identifiable. If the GSF curves of two estimated parameters exhibit opposite trends in slope, it indicates a high degree of correlation between them (Pant et al., 2014; Banks et al., 2007).

**FIGURE 3:**
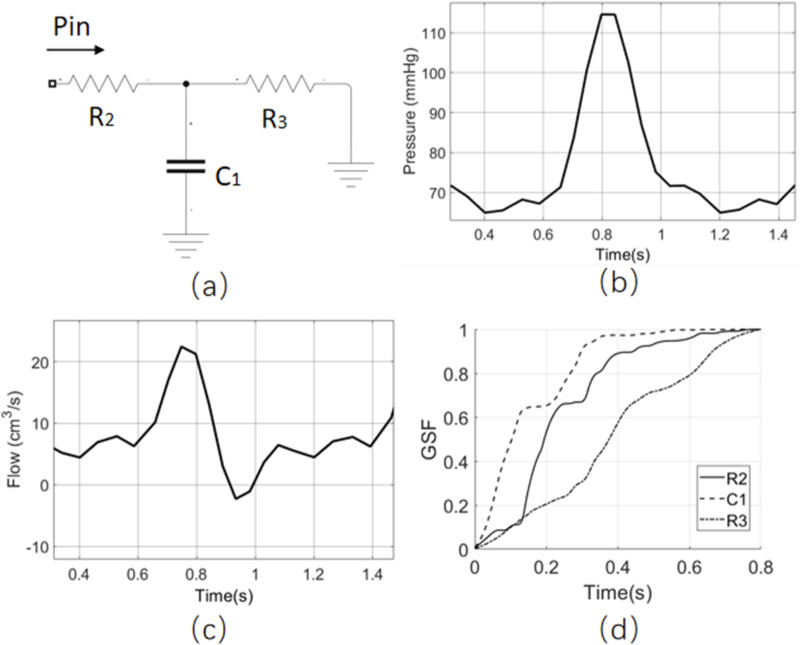
The GSF analysis of the RCR model. **(a)** The model structure of RCR, **(b)** the inlet boundary condition, **(c)** the inlet flow as the target index, **(d)** the GSF result of three parameters.

A simple resistor-capacitor-resistor (RCR) structure component is used to demonstrate how the GSF can be employed for MIA, as shown in Figure 3. The model structure is depicted in (a), with the inlet boundary condition represented by a pressure waveform with a period of 0.8 s (as shown in Figure 3b), and the outlet grounded. Using the inlet flow waveform in Figure 3c as the target index, the GSF is calculated for R2/C1/R3, with the results displayed in Figure 3d. All three curves exhibit a monotonically non-decreasing behaviour, indicating that the contributions of the target index to the three estimated parameters are relatively uniform. It suggests that there are no identifiability issues with this RCR model.

**FIGURE 4:**
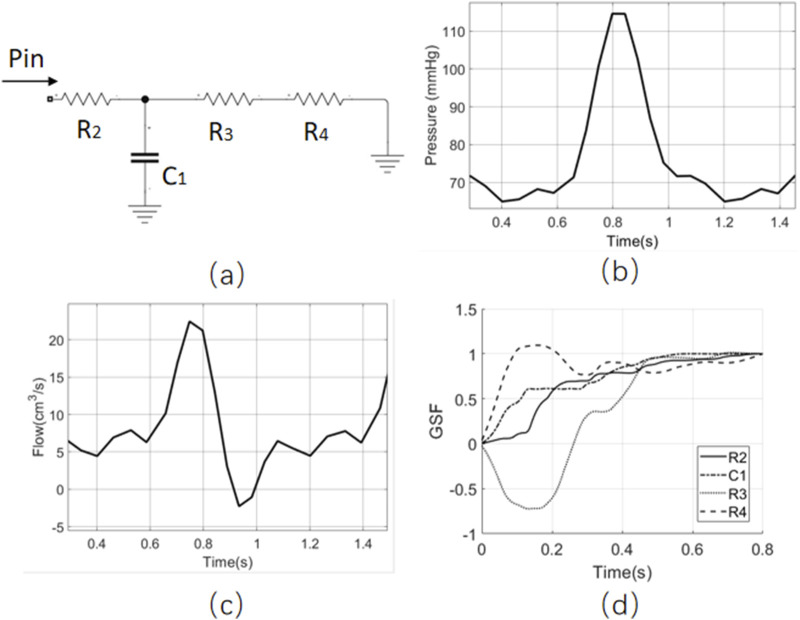
The GSF analysis of the RCRR model. **(a)** The model structure of RCRR, **(b)** the inlet boundary condition, **(c)** the inlet flow as the target index, **(d)** the GSF result of four parameters.

To further illustrate this concept, the R3 resistor of the RCR model is split into two resistors (R3 and R4) such that the new R3+R4 equals the original R3, as shown in Figure 4. The inlet and outlet boundary conditions and target index remain the same as in Figure 3. The GSF is calculated for R2/C1/R3, and the results are shown in Figure 4d. The GSF curves of R3 and R4 exhibit opposite slope trends, indicating a high correlation between R3 and R4. It suggests that this RCRR model has issues with parameter identifiability. This aligns with intuitive reasoning: based solely on the inlet flow waveform, the sum of R3 and R4 can be estimated. However, it cannot accurately determine the individual values of R3 and R4 unless the voltage between R3 and R4 is also included as a target index.

This work employs a combination of TSF and GSF for MIA. The steps are as follows: first, a TSF analysis is conducted on all parameters of the systemic and pulmonary circulation components, selecting the top N parameters with the highest TSF values as candidates; second, a GSF analysis is performed on these N candidates to identify and exclude any that exhibit identifiability issues. In this study, N=10 . Section 3.2 presents the results of the TSF and GSF analyses.

By now, the (1) to (3) categories have been introduced, as listed in Table 4. The remaining parameters in the LPM may be classified under category (4). The LVAD component, whose properties are predefined by the device and manufacturer specifications, will not undergo optimization in this study. This approach prioritises the estimation of clinically relevant parameters with diagnostic significance, while keeping other variables constant to ensure computational efficiency and model stability.

**TABLE 4:** Variables involved in dataset generation, along with their initial values and sampling ranges.

Category	Location	Parameter	Initial value	Sample range
(2)	Left ventricle	$E_{LV}^{\max}$	1.0	[−90%,+100%]
(2)	Right ventricle	$E_{RV}^{\max}$	1.15	[−90%,+100%]
(1)	Systemic circulation	$R_{\mathrm{sar}}$	1.02	[−50%,+50%]
		$R_{\mathrm{sat}}$	0.05	[−50%,+50%]
		$C_{\mathrm{sat}}$	1.0	[−50%,+50%]
		$R_{ao}$	0.003	[−50%,+50%]
(1)	Pulmonary circulation	$R_{\mathrm{pas}}$	0.002	[−50%,+50%]
		$C_{\mathrm{pvn}}$	20.5	[−50%,+50%]
(3)	Cardiac cycle	T	0.8	[−80%,+80%]
(3)	Total blood volume	$V_{\mathrm{total}}$	900	[−60%,+60%]

### 2.3 Dataset generation

This work aims to achieve patient-specific estimation of LPM parameters through CLPM-Net. Specifically, non-invasive physiological indicators (obtained from ultrasound and pressure sensors) are input into the well-trained CLPM-Net, which can output patient-specific parameters of the LPM. Clinical data best reflect the pathological characteristics of HF patients, but such data are often scarce and difficult to obtain. Therefore, synthetic data were generated through LPM for the training and testing of CLPM-Net, while three clinical cases were used for the clinical validation of CLPM-Net.

#### 2.3.1 Artificially synthesized data

**FIGURE 5:**
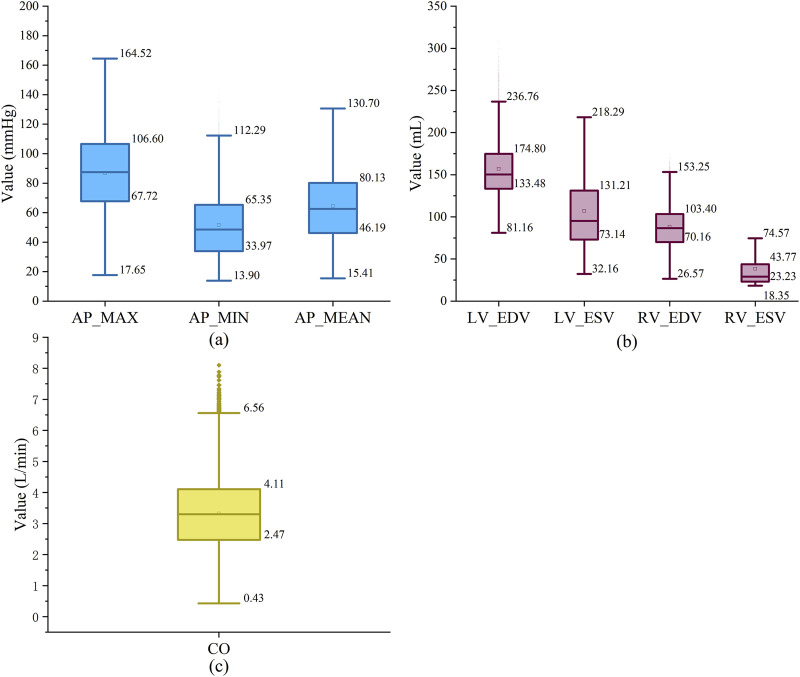
Hemodynamic index in dataset calculated by LPM with annotated minimum, maximum, 25% and 75% values. **(a)** Arteria pressure (AP_MAX, AP_MIN, AP_MEAN) in Artificially synthesized data (mmHg), **(b)** ventricular volumes (LV_EDV, LV_ESV, RV_EDV, RV_ESV; mL), **(c)** cardiac output (CO; L/min).

In the absence of clinical data, the ten variable parameters can be adjusted within a defined range based on the non-specific values (see Table 4). Table 4 outlines the initial values and sampling ranges for the ten estimated parameters. The Latin hypercube sampling method was utilised, resulting in a total of M sampling points within the designated range. In this work, M=18,000 . The 18,000 synthetic data samples were generated using the LPM of the native circulatory system (without LVAD). This ensures that the CLPM-Net focuses on learning the intrinsic cardiovascular properties of the patient, making the framework adaptable to different LVAD models during clinical application. For those LPM parameters absent in Table 4, the default values listed in Table 1 are utilised. Each of the 18,000 LPM parameter sets is simulated, thereby producing 18,000 distinct physiological indicators. Consequently, 18,000 feature-label pairs for CLPM-Net are obtained, with 14,000 pairs randomly selected for training, 2,000 for validation, and 2,000 for testing. The 18,000 sets are quantitatively evaluated to ensure they adequately encompass the physiological characteristics of all HF patients (see Figure 5).

#### 2.3.2 Clinical data

A prospective data collection was conducted at the Children’s Hospital Zhejiang University School of Medicine. As of June 2025, de-identified clinical data from three patients with dilated cardiomyopathy have been collected, as detailed in Table 5. All three clinical patients were implanted with the CorHeart 6 LVAD. After anaesthesia and before thoracotomy, all patients received supine echocardiography, right heart catheterisation and invasive monitoring. The specific device parameters from Table 2 were incorporated into the patient-specific LPM after the neural network predicted the individual’s physiological parameters.

**TABLE 5:** Information on three patients included in this study.

Patient No.	Gender	Age (years)	BSA ( ${\text{m}}^{2}$ )
Patient 1	Female	10	1.18
Patient 2	Male	10	1.20
Patient 3	Male	10	1.20

### 2.4 The architecture of CLPM-Net

**FIGURE 6:**
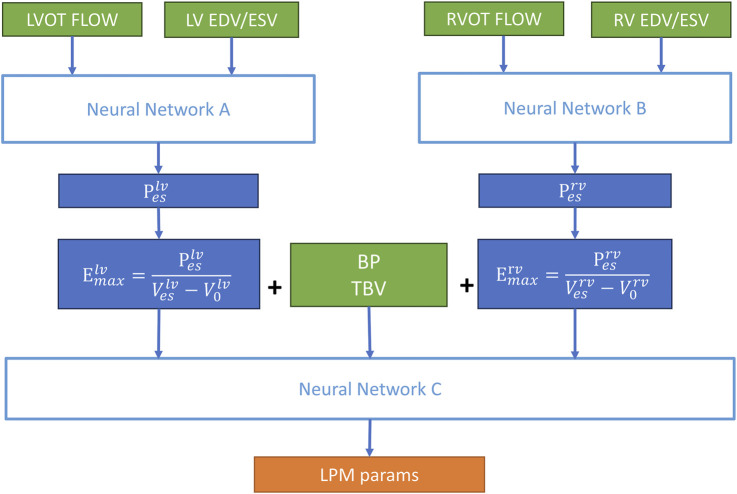
The structure of CLPM-Net. NN A and NN B predict the left and right ventricular end-systolic pressures ( $P_{es}^{lv}$ and $P_{es}^{rv}$ ) from ultrasound flow waveforms and volumes. NN C predicts the remaining LPM parameters using the outputs from NN A and NN B, along with blood pressure (BP) and total blood volume (TBV).

CLPM-Net is a hierarchical NN designed to predict LPM parameters using non-invasive physiological indicators obtained from ultrasound and blood-pressure monitors. Among the parameters listed in Table 4, the T can be directly obtained from ultrasound, and the TBV can be calculated based on height and weight. Therefore, the actual output of the CLPM-Net consists of 8 parameters. The structure of CLPM-Net is illustrated in Figure 6, and it is divided into three steps:

STEP 1: Input the physiological indicators obtained from ultrasound (outflow tract flow waveforms, end-systolic volume, and end-diastolic volume of the left and right ventricles) into Neural Networks (NN) A and B to obtain the end-systolic pressures of the left and right ventricles.

STEP 2: According to Equation 2, $E_{RV}^{\max}$ and $E_{LV}^{\max}$ are calculated. The numerator on the right side of Equation 2 comes from NN A and B. The denominator consists of the end-systolic volume measured from ultrasonography (which is also one of the inputs to NN A/B) and the default zero-pressure volume (see Table 1).

STEP 3: Input the $E_{RV}^{\max}$ and $E_{LV}^{\max}$ , non-invasive blood pressure, and TBV into NN C to obtain the eight estimated parameters.

In STEP 1, NN A and B achieve the mapping between hemodynamic parameters. From a physical perspective, the slope and amplitude characteristics of the flow waveform reflect the dynamic response of the heart and the LVAD working together against the afterload Imamura et al. (2018). A wide sampling range ( ± 50% to ± 90%) also ensures that the training dataset covers the most common physiological states. STEP 2 utilises physical constraints to map the hemodynamic indicators (the right side of Equation 2) to the LPM parameters (the left side of Equation 2). STEP 2 is a bridge to connect the hemodynamic indicators and LPM parameters. After completing STEPs 1 and 2, the patient-specific optimizations related to the heart component are finished. STEP 3 focuses on optimizing the parameters of the systemic and pulmonary circulation components. The blood-pressure waveform and TBV contain high-dimensional information about the resistance, elasticity, and inertia of the systemic and pulmonary circulation, which can be extracted through NN C. Ultimately, it outputs the corresponding LPM parameters for the systemic and pulmonary circulation components.

**FIGURE 7:**
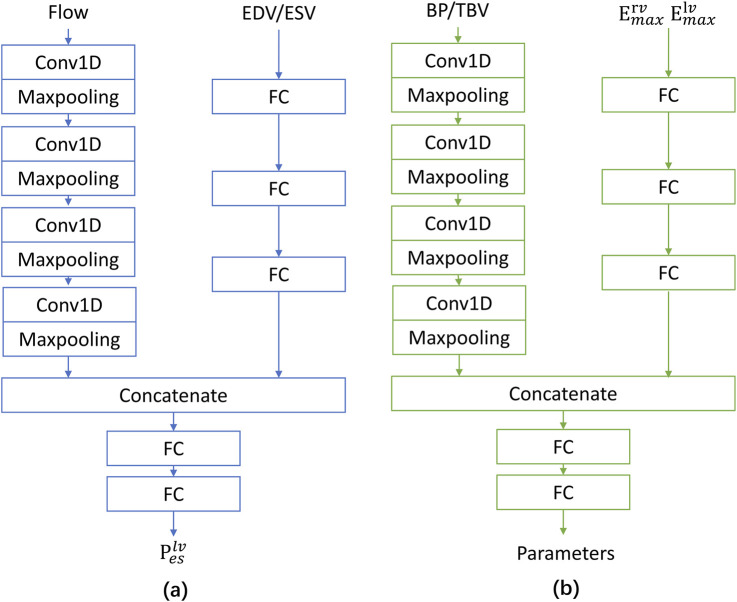
**(a)** Network architecture with inputs of flow and EDV/ESV, producing P^lv^_es_ as output. **(b)** Network architecture with inputs of BP/TBV, E^rv^_max_, E^lv^_max_, producing model parameters as output.

NN A/B/C have the same structure (see Figure 7), but they are independent and do not share parameters. A hierarchical structure can significantly enhance the patient-specificity optimization capability for capturing highly nonlinear characteristics of LPM. It combines the physics-constrained (STEP 2) and data-driven (STEP 1 and 3) approaches.

Here are some additional details about CLPM-Net. The loss function used is mean squared error (MSE). The learning rate is set to 0.001. To prevent overfitting, an early stopping strategy is employed. If the loss of the current epoch is lower than that of the previous epochs, the current model parameters are saved as the “best” model. All training and testing are conducted on the PyTorch platform (version 2.1.0), using an NVIDIA GeForce RTX 4090 GPU.

Each network of CLPM-Net is a dual-branch model: one branch processes 1D signal inputs through a four-layer convolutional neural network (CNN) with progressively increasing channel depths (2 → 16 → 64 → 256 → 512), kernel size of 3, ReLU activations, and max-pooling after each layer; the other branch maps structured features via a multilayer perceptron (MLP) with three hidden layers of 64 units and ReLU activations. The outputs from both branches are flattened, concatenated, and passed through a classifier consisting of fully connected layers (1,024 → 64 → 1 for NN A/B and 1,024 → 64 → 6 for NN C). The architecture is tailored specifically for patient-specific optimization in heart failure rather than as a general-purpose model, ensuring focused adaptability to individual physiological signatures.

## 3 Results

### 3.1 Results of basic lumped parameter model

**FIGURE 8:**
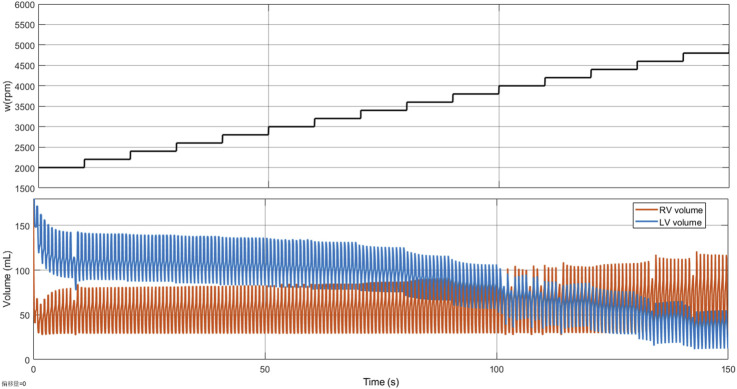
The results of the ramp test for the basic LPM. The subgraph above shows the LVAD rotation speed, and the subgraph below shows the right ventricle (RV) volume and left ventricle (LV) volume. These two subgraphs share the same x-axis.

The basic LPM in Section 2.1 is validated through a simulated ramp test with the HeartMate 3, ensuring consistency with the clinical ramp test (Addetia et al., 2018). Figure 8 illustrates how the left ventricular and right ventricular volumes change with increasing rotational speed. The LPM parameters used in Figure 8 are from Table 1 and $E_{LV}^{\max}=1.0$ . The ramp test is conducted initially by LVAD backflow and concludes with suction. As shown in Figure 8, the left ventricular volume gradually decreases while the right ventricular volume gradually increases. The volume decrease in the left ventricle is twice that of the volume increase in the right ventricle. These observations are consistent with clinical findings from (Addetia et al., 2018; Uriel et al., 2019).

**FIGURE 9:**
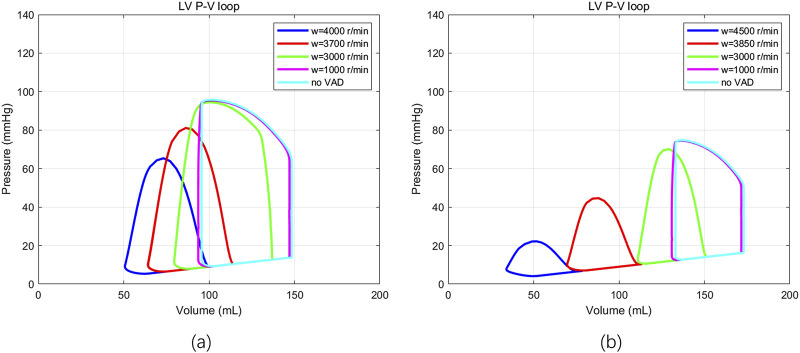
The pressure-volume loop observed in two patients with HF in response to variations in the LVAD rotation speed. **(a)** Patients with mild HF ( $E_{\max}^{LV}$ = 1.0), **(b)** patients with severe HF ( $E_{\max}^{LV}$ = 0.6).

In addition, the pressure-volume loops were also evaluated (see Figure 9) for HF patients with two different severities under varying rotational speeds of the HeartMate 3. It can be observed that as the LVAD rotational speed gradually increases, the pressure-volume loop shifts towards the lower left corner. Its shape transitions from a trapezoid to a triangle. This is consistent with the conclusions drawn by Giridharan et al. (2015), and Park et al. (2018). This verifies the accuracy of the basic LPM introduced in Section 2.1 by utilising results of the ramp test and the pressure-volume loops.

### 3.2 Results of identification analysis

**FIGURE 10:**
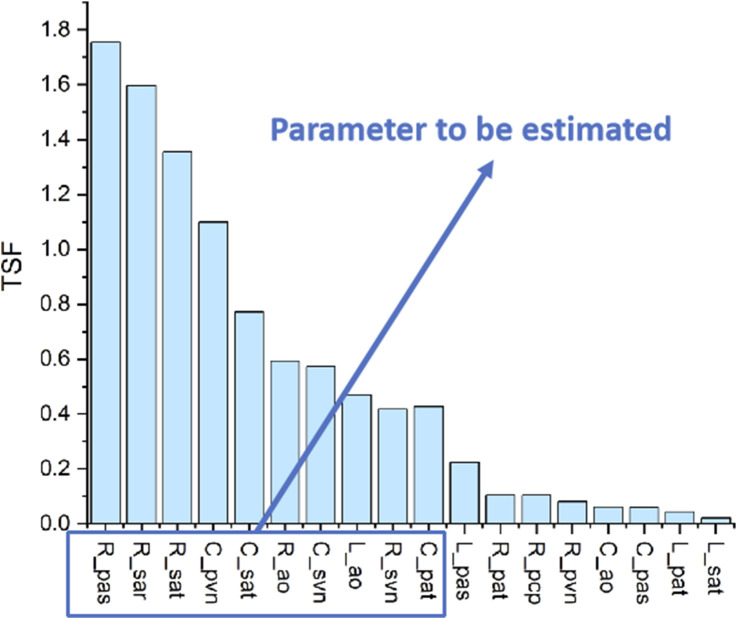
The results of traditional sensitivity analysis.

**FIGURE 11:**
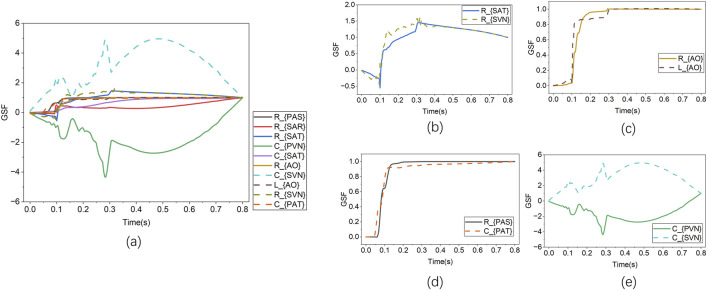
The results of global sensitivity analysis. **(a)** GSF analysis across all ten model parameters. **(b**–**e)** GSF comparisons between four selected parameter pairs.

Section 2.2 introduces two methods for MIA: TSF and GSF. Figure 10 presents the TSF results for all systemic and pulmonary circulation parameters concerning CO/AP/PAP. In particular, the TSF values of each LPM parameter to CO/AP/PAP are first calculated (as shown in Equation 4), followed by normalisation and summation. The top N parameters with the highest TSF values are selected for the subsequent GSF analysis, with N=10 . The GSF curves for these ten parameters are then plotted over one cardiac cycle to examine whether there are any identifiability issues among the ten parameters. According to the results in Figure 11, the GSF curves of $R_{\mathrm{sat}}$ and $R_{\mathrm{svn}}$ , $R_{ao}$ and $L_{ao}$ , and $R_{\mathrm{pas}}$ and $C_{\mathrm{pat}}$ are closely correlated, indicating strong pairwise correlations. Meanwhile, the curves of $C_{\mathrm{pvn}}$ and $C_{\mathrm{svn}}$ exhibit opposite trends, confirming an identifiability issue. To resolve these identifiability conflicts, the parameters $R_{\mathrm{svn}}$ , $L_{ao}$ , $C_{\mathrm{pat}}$ , and $C_{\mathrm{svn}}$ , which have relatively lower TSF values, were excluded from the set of parameters to be estimated. The remaining six parameters constitute the final set for patient-specific optimization. All other parameters in the model are held constant at the values specified in Table 1. These six parameters are thoroughly analyzed and confirmed to have the greatest impact on model behavior. Given the limited clinical data, retaining this limited set helps control computational cost and avoids introducing unnecessary noise or uncertainty. To emphasize the potential impact of fixing other parameters, the validity of this model will be based on the existing dataset. The results also indicate that no MIA issues remain.

The evaluation metric of CLPM-Net is the Root Mean Square Error (RMSE), as shown in Equation 8. Here, n represents the number of cases, $y_{i}$ is the predicted value, and ${\hat{y}}_{i}$ is the ground truth value. Table 6 presents the prediction errors for two heart parameters and six systemic and pulmonary circulation parameters. $E_{\max}^{LV}$ and $E_{\max}^{RV}$ are derived from STEP 2 of CLPM-Net, with the pressure values coming from NN A/B. The six systemic and pulmonary circulation parameters are directly output by NN C. The errors inherent in $E_{\max}^{LV}$ and $E_{\max}^{RV}$ will accumulate in the output of C, which will be discussed in the next section. Despite the limitation of error accumulation, the prediction errors for the six pulmonary circulation parameters remain below 10%.

$$RMSE=\sqrt{\frac{1}{n}\overset{n}{\underset{i=1}{\sum }}{\left(y_{i}-{\hat{y}}_{i}\right)}^{2}}.$$ (8)

**TABLE 6:** The direct output of CLPM-Net and the corresponding errors. RMSE: Relative mean square error.

Calculated by	Output	RMSE
Equation 2	$E_{\max}^{LV}$	9%
Equation 2	$E_{\max}^{RV}$	7%
Neural network C	$R_{\mathrm{pas}}$ / $R_{\mathrm{sar}}$ / $R_{\mathrm{sat}}$	4.39%/8.85%/2.25%
	$C_{\mathrm{pvn}}$ / $C_{\mathrm{sat}}$ / $R_{ao}$	1.59%/0.448%/9.57%

**FIGURE 12:**
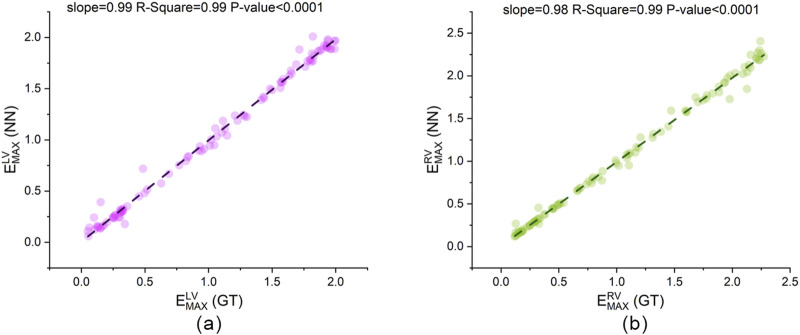
The linear regression plot of CLPM-Net’s output and the ground truth. **(a)** $E_{\max}^{LV}$ and **(b)** $E_{\max}^{RV}$ , GT: ground truth from synthetic data, NN: neural network, that is CLPM-Net.

**FIGURE 13:**
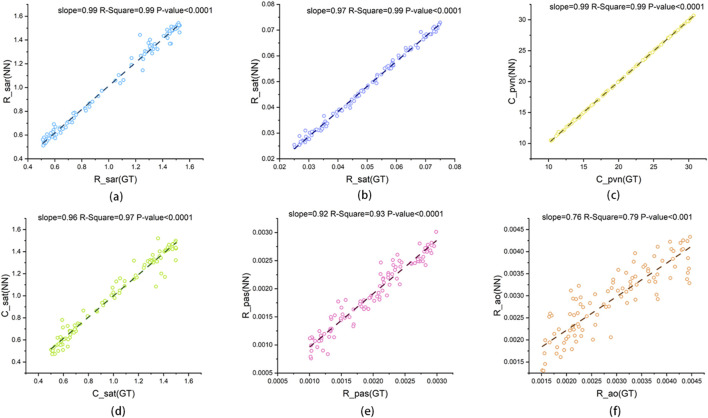
The linear regression plot of CLPM-Net’s output and the ground truth for the six systemic and pulmonary circulation parameters: **(a)** R_sar (GT), **(b)** R_sat (GT), **(c)** C_pvn (GT), **(d)** C_sat (GT), **(e)** R_pas (GT) and **(f)** R_ao (GT), GT: ground truth from synthetic data, NN: neural network, that is CLPM-Net.

Figures 12, 13 present scatter plots of the predicted values and ground truth values for two cardiac parameters and six systemic and pulmonary circulation parameters. For $E_{\max}^{LV}$ and $E_{\max}^{RV}$ , the slopes of the fitted lines for all scatter points are very close to 1 (slope = 0.99 and 0.98), and the scatter points are concentrated near the fitted lines (R-square = 0.99). For the six parameters in Figure 13, all fitted lines are close to 1 (slope = 0.92–0.99) except for $R_{ao}$ . This indicates a good consistency between the predicted values and the true values. The prediction performance for $R_{ao}$ is slightly inferior to that of the other parameters, which may be due to the smaller magnitude of its true values, leading CLPM-Net to capture its nonlinear characteristics less effectively than the other parameters. Another possible reason is that the TSF curve for $R_{ao}$ converges to 1 earlier, resulting in insufficient information contribution from the latter half of the cardiac cycle. However, its quantitative error remains within 10% (RMSE = 9.57%). Therefore, there is ample reason to believe that CLPM-Net can serve as an equivalent substitute for inverse LPM calculations.

### 3.3 Results of CLPM-Net

**FIGURE 14:**
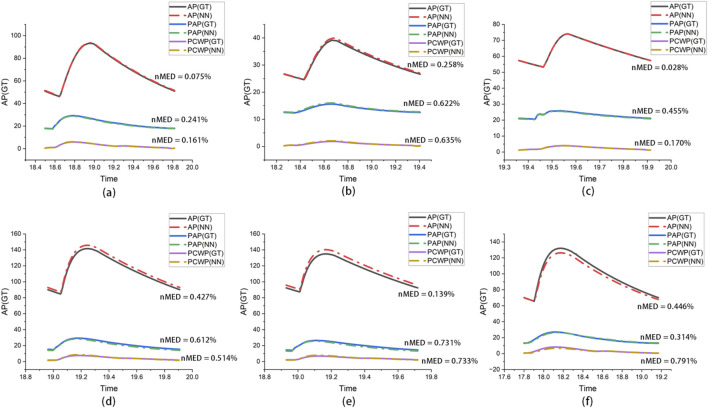
The comparative NN prediction results of the AP, PAP, and PCWP with the selection of the six specific parameters. **(a–c)** Three best-performing test samples and **(d–f)** three worst-performing samples.

In addition to evaluating the accuracy of the eight specific LPM parameters directly output by CLPM-Net, the accuracy of the hemodynamic indicators obtained after running the specific LPM was also assessed. Figure 14 shows the three samples with the best performance and the three samples with the worst performance in the test set. For each sample, three indicators were selected: AP, PAP, and PCWP for assessment. Dashed lines represent the values calculated by CLPM-Net, while solid lines represent the ground truth. The error between the CLPM-Net results and the ground truth is quantitatively assessed using the Euclidean distance (ED) as defined in Equation 9. To achieve normalization and improve comparability, the normalized mean Euclidean distance (nMED, Equation 10) was further introduced. Validation experiments of nMED has been performed, see Appendix A.

$$ED=\overset{N}{\underset{i=1}{\sum }}\sqrt{{\left(x_{gt,i}-x_{\mathrm{pred,i}}\right)}^{2}+{\left(y_{gt,i}-y_{\mathrm{pred,i}}\right)}^{2}}\;,$$ (9)

$$nMED=\frac{ED}{N\times \left(y_{gt,max}-y_{gt,min}\right)}\;.$$ (10)

In the above equations, i represents different time steps, $\left(x_{\mathrm{pred}},y_{\mathrm{pred}}\right)$ represents the predicted values, and $\left(x_{gt},y_{gt}\right)$ represents the ground truth values. In both the three best-performing and the three worst-performing samples, the predicted values and the ground truth are visually very similar. Quantitatively, nMED is less than 1% across all six cases (0.028%–0.791%).

**FIGURE 15:**
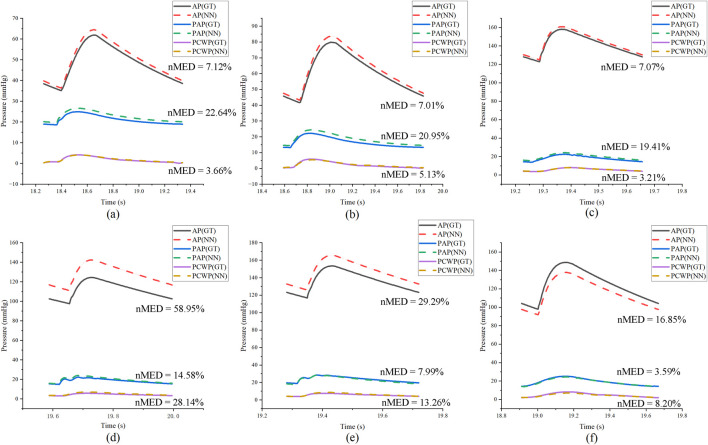
The comparative NN prediction results of the AP, PAP, and PCWP with the selection of the ten specific parameters. **(a–c)** Three best-performing test samples and **(d–f)** three worst-performing samples.

To emphasize the rationale behind selecting the six LPM parameters in Section 3.2, the accuracy of AP, PAP, and PCWP was also evaluated using the top ten LPM parameters based on TSF results. Figure 15 shows the three best-performing and three worst-performing samples in the test set. After selecting the top ten parameters as the patient-specific optimization parameter set, the nMED significantly increased to 3.21%–58.95%. These results demonstrate that fixing the other parameters helps improve the model’s accuracy.

## 4 Discussion

### 4.1 Impact of hierarchical structure on results

One of the key innovations of this work is the proposal of a hierarchical structure (NN A/B/C). This design preserves the structural complexity of the 12-compartment LPM, representing an advantage over PINNs. NN A/B initially predicts the hemodynamic parameters of the cardiac component using data-driven approaches, and then applies physical constraints (Equation 2) to transform these hemodynamic parameters into LPM parameters. Following this, NN C predicts the LPM parameters for the systemic and pulmonary circulation components, also based on data-driven methods. As shown in Section 3.3, this structure enables effective simulation of the parameters.

**FIGURE 16:**
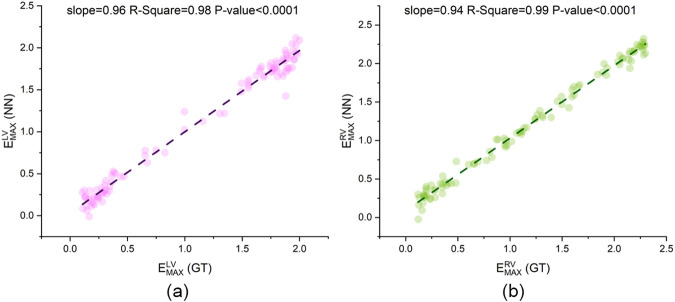
The linear regression plot of non-hierarchical NN’s output and the ground truth. **(A)** $E_{\max}^{LV}$ and **(B)** $E_{\max}^{RV}$ , GT: ground truth from synthetic data, NN: non-hierarchical neural network.

**FIGURE 17:**
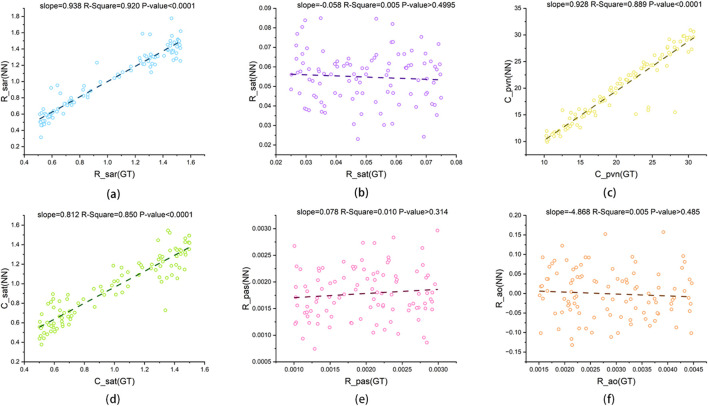
The linear regression plot of non-hierarchical NN’s output and the ground truth for the six systemic and pulmonary circulation parameters: **(a)** R_sar (GT), **(b)** R_sat (GT), **(c)** C_pvn (GT), **(d)** C_sat (GT), **(e)** R_pas (GT) and **(f)** R_ao (GT), GT: ground truth from synthetic data, NN: non-hierarchical neural network.

To further validate the significance of the hierarchical structure and physical constraints, a NN for comparative validation against CLPM-Net was designed: NN A/B and Equation 2 were removed, leaving only NN C. This network serves as the baseline for comparison against our proposed CLPM-Net. It was fed the same inputs of CLPM-Net, corresponding to the content in the green box of Figure 6. The output of this modified network matches that of CLPM-Net. The results are presented in Figures 16, 17.

With NN A/B and Equation 2 retained, CLPM-Net achieved high agreement across all eight parameters. Using ground truth as the x-axis and predictions as the y-axis, seven parameters had fitted-line slopes close to 1 (0.92–0.99; see Figures 12, 13). The remaining parameter $R_{ao}$ showed slightly inferior performance due to its smaller magnitude and the early convergence of its TSF curve, yet its quantitative error was still below 10% (RMSE = 9.57%). After removing the hierarchical structure (Figures 16, 17), the two cardiac parameters still agreed reasonably with ground truth (slopes = 0.96 and 0.94, slightly below 0.99 and 0.98 before removal). However, the prediction performance for the systemic and pulmonary circulation parameters are relatively poor. In particular, the fitted-line slopes for $R_{\mathrm{sat}}$ , $R_{\mathrm{pas}}$ , and $R_{ao}$ collapsed from 0.97 to 0.058, from 0.92 to 0.078, and from 0.76 to −4.868 , respectively. This indicates that this simplified NN is completely unable to estimate these three parameters.

These results indicate that removing NN A/B and Equation 2 when simulating parameters causes large discrepancies from patient reality and prevents construction of a patient-specific LPM, highlighting the critical importance of the proposed hierarchical structure and physical constraints in CLPM-Net. This also demonstrates that end-systolic pressure acts as a bridge, enabling the model to decouple and extract afterload information from non-invasive flow waveforms.

### 4.2 Clinical validation

**FIGURE 18:**
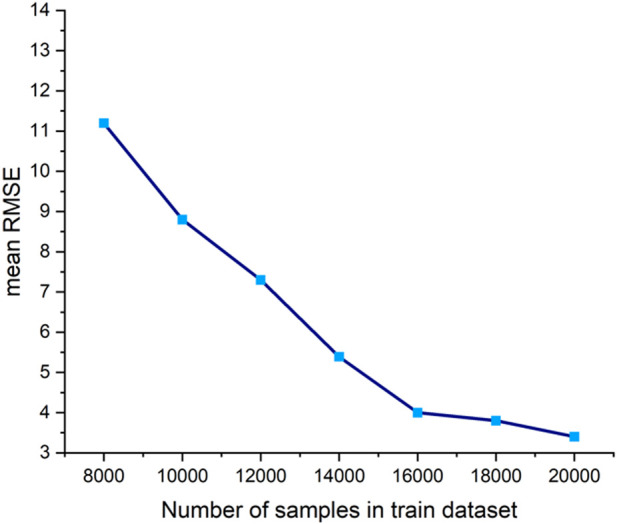
The relationship between prediction error and the size of the train dataset as it increases.

Given the scarcity and difficulty of obtaining real clinical data, the training set, test set, and validation set for CLPM-Net were all derived from the synthetic LPM dataset (see Table 4). Consequently, the current model has only been validated within the distribution range of the training data. The impact of training set size on the performance of CLPM-Net is also explored, as shown in Figure 18. It can be observed that as the size of the training set increases, the prediction error of CLPM-Net on the same test set gradually decreases.

These results demonstrate that the model can achieve stable and accurate predictions when trained on sufficiently large synthetic datasets. To further assess its practical applicability, we conducted preliminary clinical validation. The clinical data in Section 2.3.2 were used as input, and CLPM-Net was employed to calculate the patient-specific LPM parameters. If the hemodynamic indicators calculated by the patient-specific LPM align with the results from the invasive measurement, this method could be considered as a potential alternative to invasive hemodynamic monitoring.

As shown in Table 7, the results of the invasive measurements for the three cases are quite close to the results obtained from our algorithm. Notably, CVP, which reflects right-sided preload and venous return, performed best. The predicted ranges of CVP fell entirely within invasive reference ranges, with a maximum absolute difference of 2 mmHg, and patient B showed perfect concordance. PAP showed comparably strong agreement (perfect concordance in patient B; no invasive reference available for patient A). Although patient C exhibited slight fluctuations, the values still overlapped closely with the invasive reference ranges. CO deviations were 0.2–0.4 L/min, and the predicted ranges for AP overlapped extensively with the invasive ranges. This indicates that our non-invasive algorithm has the potential to serve as a substitute for invasive hemodynamic monitoring.

**TABLE 7:** Clinical validation results for three patients.

Patient	Measurement	CVP (mmHg)	CO (L/min)	AP (mmHg)	PAP (mmHg)
Patient A	Invasive measured	[4,6]	1.1	[118,134]	–
	This work calculated	[3.5,5]	1.3	[120,132]	–
Patient B	Invasive measured	7	2.2	[99,120]	4
	This work calculated	7	2.4	[100,128]	4
Patient C	Invasive measured	[10,12]	1.1	[48,77]	[8,25]
	This work calculated	[9,11]	1.5	[45,69]	[10,20]

### 4.3 Clinical significance

This study proposed a framework that maps noninvasively acquired echocardiographic and blood-pressure indices to a set of patient-specific LPM parameters, demonstrating a potential computational pathway toward hemodynamic management in LVAD care. By inputting echocardiographic and blood-pressure indices into the hierarchical NN, the model infers patient-specific LPM parameters in real time and thus simulates key hemodynamic metrics (CO, AP and PAP) for perioperative clinical monitoring in LVAD-implanted patients. First, LVAD management depends heavily on invasive RHC, making frequent hemodynamic surveillance costly and impractical. As a result, patients living long-term with an LVAD often experience a range of adverse outcomes. Excessive LVAD pump speed can lead to over-unloading of the left ventricle, diminished right-heart preload with oscillatory cardiac output, and an elevated thrombotic risk due to low-shear/flow stagnation states. In contrast, insufficient pump speed may fail to achieve adequate left-ventricular unloading, thereby promoting persistent left-sided filling pressures and the development of secondary pulmonary hypertension (PH). Therefore, the proposed framework provides a basis for exploring noninvasive approaches to hemodynamic assessment, with the long-term aim of facilitating early identification and timely correction of potential adverse hemodynamic disturbances. Secondly, the derived patient-specific LPM parameters can be used for preoperative LVAD surgical simulation. Although LPM cannot resolve 3D flow resolution and thus cannot directly assess LVAD outflow-graft orientation or anastomosis site, the output it provides can be used to infer postoperative risk profiles. When necessary, imaging can be integrated to conduct targeted validation of the flow field. Third, this framework could be adapted to support longitudinal assessment of post-LVAD right-ventricular afterload and systemic perfusion, as well as serve as a platform for noninvasive ramp testing. These functions could inform real-time optimization of pump speed and medications, helping to avert adverse hemodynamic disturbances. Failure to achieve timely adjustments may lead to excessive right heart load, insufficient perfusion, or reduced CO. These will potentially exacerbate the patient’s symptoms and lead to severe complications, such as pulmonary hypertension, impaired organ perfusion, or worsening HF.

### 4.4 Limitations and future work

Owing to the limited number of clinical cases, the clinical validation in this study remains modest. As the model was mainly trained on synthetic data generated by the LPM, future studies should include multi-center clinical cohorts to provide prospective, real-world validation. Furthermore, considering that clinical data remain scarce and difficult to obtain, future work could incorporate multifidelity testing (Meng and Karniadakis, 2020; Mehdi et al., 2025). Synthetic data would be treated as low-fidelity data, while clinical data would be considered high-fidelity data. After training the model with low-fidelity data, the model could be further trained and validated using high-fidelity data to improve its accuracy.

Moreover, the proposed framework is built on LPM, a 0D model which captures temporal dynamics but not spatial distributions. Therefore, while this framework confers computational efficiency, it lacks spatial detail and is not suited for direct analysis of 3D cardiovascular loads, such as stresses acting on the aortic valve. Besides, the current CLPM-Net was primarily trained on noise-free synthetic data. In future research, noise injection (e.g., Gaussian noise) into the synthetic training datasets as a form of data augmentation will be used to further enhance the model’s robustness and generalization across diverse clinical scenarios.

Additionally, patients with HF may develop secondary pulmonary hypertension (Feng et al., 2024; Fang et al., 2012; Sparrow et al., 2018). Under normal physiology, RV ejects deoxygenated venous blood into the pulmonary circulation with each beat. Accordingly, the pulmonary vasculature must exhibit high compliance to accommodate both the cyclic tethering imposed by right-ventricular ejection and the oscillatory mechanical tethering associated with bronchial and alveolar inflation–deflation. Under pathological conditions, the pulmonary circulation operates under sustained high transmural pressure. The pulmonary arterial wall is subjected to elevated circumferential wall stress (CWS) and transmural wall stress (TWS), accompanied by spatially heterogeneous and oscillatory wall shear stress (WSS) and its gradients. At vascular bends or bifurcations, secondary flows or recirculation zones are more likely to form. Additionally, pulmonary vascular cells sense these mechanical cues and activate signaling pathways via mechanotransduction. This drives structural remodeling, including wall thickening and fibrosis. In rare high-risk scenarios, dissection-like changes of the pulmonary artery may occur. Collectively, these processes manifest as reduced pulmonary vascular compliance and increased resistance (Wang et al., 2021; Delgado et al., 2007; Jackson et al., 2022).

Consequently, to capture spatial distributions that the 0D model cannot resolve, subsequent work will introduce a 0D–3D coupled framework, using the LPM outputs as inlet and outlet boundary conditions for the 3D model. Regarding secondary PH, future efforts will examine whether patient-specific hemodynamic outputs from the framework can prospectively predict disease development. Moreover, the combination of unloaded-geometry reconstruction and non-uniform wall-thickness modeling, using the second principal stress (SPS) as the risk metric, can be leveraged for early detection of structural instability (Liu et al., 2025).

## 5 Conclusion

Using data obtained from ultrasound and blood pressure, this work presents a hierarchical neural network that combines physics-constrained and data-driven approaches to enable noninvasive simulation of patient-specific LPM.

The framework was constructed on the basis of a basic LPM incorporating an implanted LVAD. In Section 3.1, the basic LPM was validated using ramp tests and pressure–volume loop assessments. These results demonstrated its ability to accurately simulate the basic physiological conditions of patients with an implanted LVAD. In Section 3.2, MIA was conducted to identify six key parameters that have the greatest influence on the LVAD-related hemodynamic outputs (CO, AP, and PAP) and to ensure that these parameters can be uniquely identified within the model.

Meanwhile, the framework incorporated a hierarchical NN, CLPM-Net, which introduced both the hierarchical structure and the physics-constrained mechanisms. After introducing the inputs, the hierarchical NN estimated two heart parameters and six systemic and pulmonary circulation parameters. The predictions exhibited strong consistency with the true values, achieving RMSE values below 10%. Moreover, the deviation between the true values and the predicted results was consistently below 1% in terms of nMED, demonstrating a high level of agreement. Therefore, CLPM-Net can serve as an effective tool for estimating patient-specific LPM parameters.

Additionally, to verify the necessity of the hierarchical structure and physics-constrained mechanisms, an ablation experiment was designed. The results indicated that after removing both the hierarchical structure and the physics-constrained mechanisms, the simplified network failed to accurately estimate the systemic and pulmonary circulation parameters.

In summary, the proposed framework has been validated through foundational LPM verification and MIA. Preliminary clinical comparisons further indicate its potential as an alternative to invasive hemodynamic monitoring. Compared with conventional invasive approaches that rely on catheterization or surgical procedures, this framework estimates patient-specific hemodynamic parameters solely from routine data (ultrasound and blood-pressure measurements), thereby obviating additional instrumentation or surgical intervention and substantially reducing risk and cost. The framework provides quantitative guidance for preoperative LVAD assessment, early and long-term postoperative follow-up, outpatient pump-speed optimization, and adjunctive pharmacotherapy. By doing so, it helps reduce hemodynamic dysregulation and complications arising from suboptimal management. Future research should prioritize large-scale clinical trials and explore integration with higher-dimensional fluid and wall mechanics models as well as imaging modalities, to accelerate clinical translation and enhance early-warning capabilities for complications such as PH.

## Data Availability

The raw data supporting the conclusions of this article will be made available by the authors, without undue reservation.
