# Supplementary material for: Patient-specific hemodynamic simulation for left ventricular assist device via non-invasive monitoring based on lumped parameter model and hierarchical neural network
Source: Front Physiol. 2026 Apr 7;17:1738196. doi: 10.3389/fphys.2026.1738196 (PMC13095542; doi:10.3389/fphys.2026.1738196)
Supplement: Supplementary file 1 [file Supplementaryfile1.pdf]

Supplementary Material

1 VALIDATION OF THE APPROPRIATENESS OF NMED

To quantitatively evaluate the similarity between predicted and ground-truth waveforms, this study employs the normalized mean Euclidean distance (nMED). Because the dataset covers a wide pathophysiological range, the Euclidean distance (ED) is influenced by both amplitude scale and the number of sampling points. nMED removes scale and sampling effects through normalization, dividing ED by the product of the waveform's range and the number of sampling points.

To validate the appropriateness of using nMED in this context, two functions were randomly generated in this study, which served as the Ground Truth (GT) waveform and the predicted (Pred) waveform. These functions were then analyzed under different scaling and sampling conditions, with the process repeated multiple times. Fig. S1 presents the ED and nMED values for the GT and Pred waveforms under different scaling conditions, while Fig. S2 presents these values under different sampling size conditions. For better visualization, only three pairs of randomly generated functions are displayed here.

In Fig. S1, we applied 25%, 50%, and 100% scaling to the two randomly generated waveforms and plotted the corresponding ED and nMED values. We observed that the ED values varied significantly with different scaling factors, which is consistent with the fact that waveform range influences the ED values. However, the nMED values remained stable across all scaling factors. This demonstrates that nMED successfully eliminates the impact of absolute scale differences through its normalization design.

In Fig. S2, we calculated the ED and nMED values for the two randomly generated waveforms with 20, 50, 100, and 200 sampling size. The ED values exhibited significantly larger fluctuations compared to the nMED values, showing that nMED is less sensitive than ED. This can be attributed to the design of the nMED formula, which divides by the number of sampling points (N), effectively mitigating the cumulative error bias caused by different sampling densities.

As for the impact of time scale, in this study, the waveforms were generated under a unified time scale and simulation step size, with strict alignment of the time references. Therefore, waveform comparisons within this framework do not suffer from time misalignment issues.

To summarize, in this study, the use of nMED to assess waveform matching is appropriate.

1.1 Figures

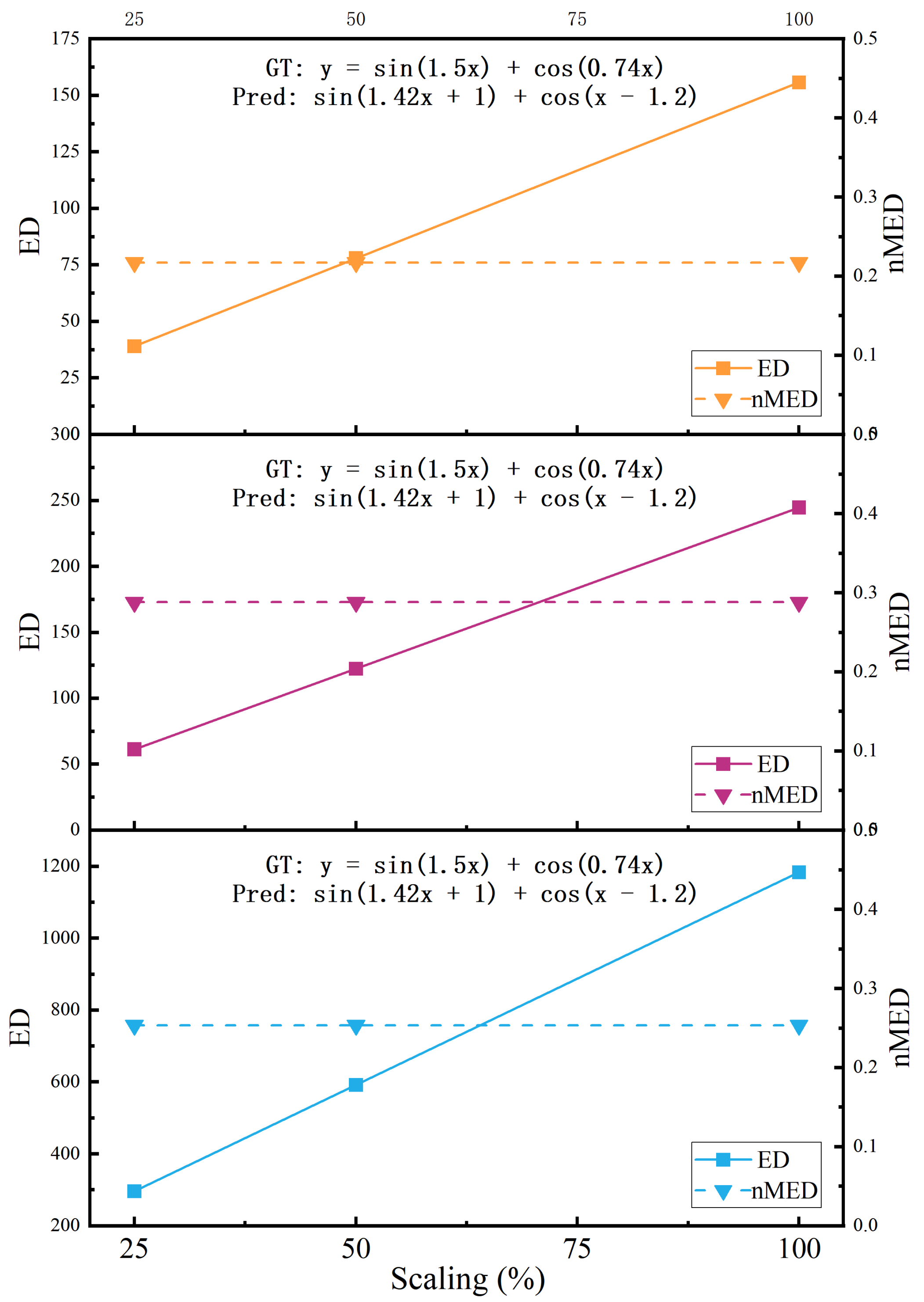

Figure S1. ED and nMED comparison under scaling 25%, 50%, 100%. GT: ground truth waveform, Pred: predicted waveform.

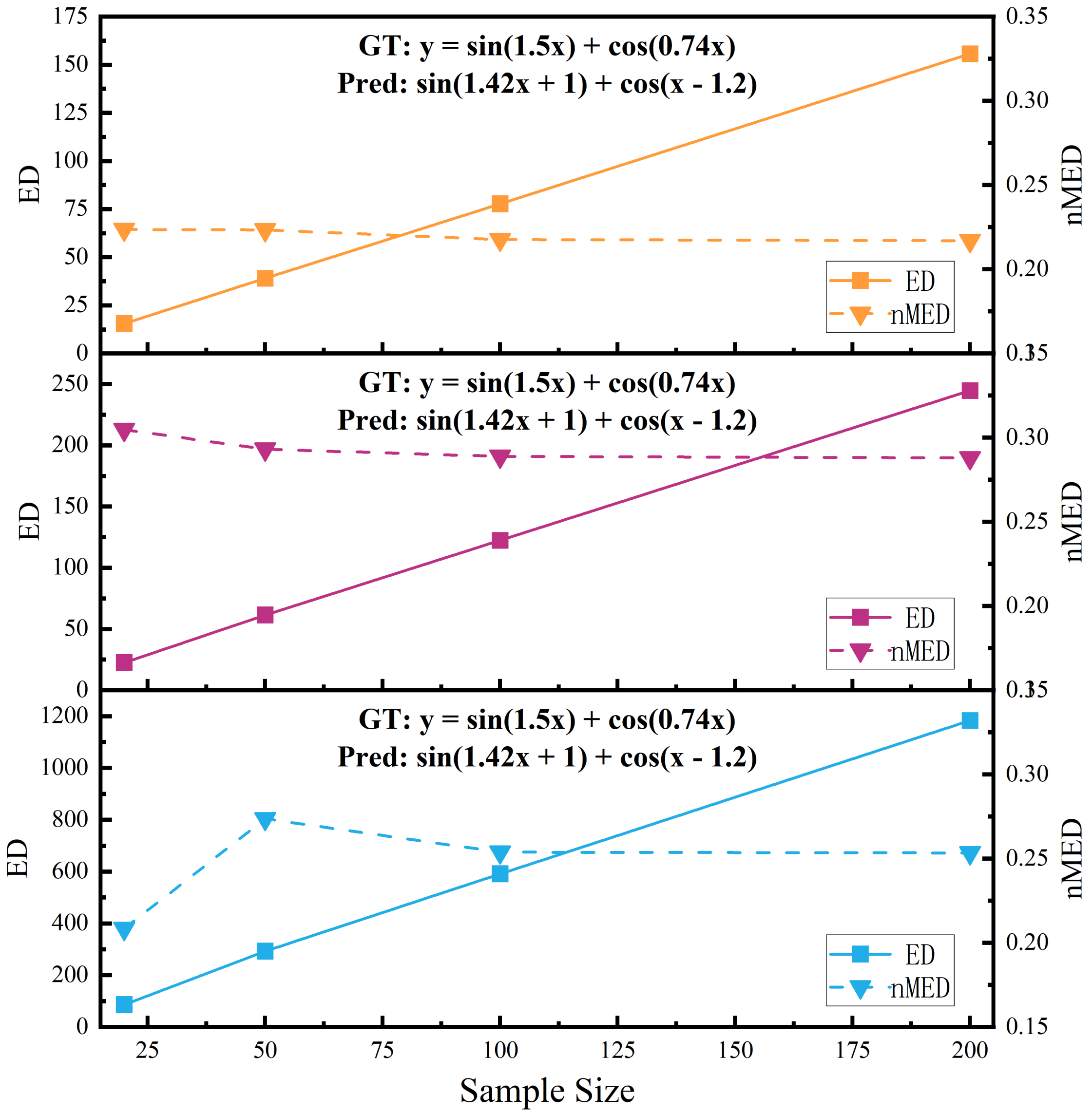

Figure S2. ED and nMED comparison under sample size 20, 50, 100, 200. GT: ground truth waveform, Pred: predicted waveform.
